# Identification of a novel large deletion and other copy number variations in the *CFTR* gene in patients with Cystic Fibrosis from a multiethnic population

**DOI:** 10.1002/mgg3.645

**Published:** 2019-06-14

**Authors:** Raisa da Silva Martins, Mario Campos Junior, Aline dos Santos Moreira, Verônica Marques Zembrzuski, Ana Carolina Proença da Fonseca, Gabriella de Medeiros Abreu, Pedro Hernan Cabello, Giselda Maria Kalil de Cabello

**Affiliations:** ^1^ Human Genetics Laboratory, Oswaldo Cruz Institute Oswaldo Cruz Foundation Rio de Janeiro Brazil; ^2^ Laboratory of Functional Genomics and Bioinformatics, Oswaldo Cruz Institute Oswaldo Cruz Foundation Rio de Janeiro Brazil; ^3^ Genetics Laboratory Grande Rio University (UNIGRANRIO) Rio de Janeiro Brazil; ^4^ Molecular and Cellular Biology Graduate Program (PPGBMC) Federal University of the State of Rio de Janeiro (UNIRIO) Rio de Janeiro Brazil

**Keywords:** CFTR, CNV, Cystic Fibrosis, MLPA, Mutation

## Abstract

**Background:**

Cystic fibrosis (CF) is caused by mutations in the cystic fibrosis transmembrane conductance regulator gene (*CFTR*). There are over 2000 different pathogenic and non‐pathogenic variants described in association with a broad clinical heterogeneity. The most common types of mutations in this gene are single nucleotide substitutions or small deletions and insertions. However, large rearrangements, such as large duplications or deletions, are also a possible cause of CF; these variations are rarely tested in routine screenings, and much of them remain unidentified in some populations, especially those with high ethnic heterogeneity.

**Methods:**

The present study utilized the Multiplex Ligation‐dependent Probe Amplification (MLPA) technique for the detection of duplications and deletions in 165 CF patients from the Rio de Janeiro State (Brazil), which after extensive mutational screening, still exhibited one or two unidentified CF alleles.

**Results:**

Five patients with alterations in MLPA signals were detected. After validation, we identified three copy number variations, one large duplication (CFTRdup2‐3) and two large deletions (CFTRdel25‐26 and CFTRdel25‐27‐CTTNBP2). Two detected deletions were not validated. They were false positives caused by a small deletion of 18 base pairs (232del18) and a point mutation (S168L) in the probe binding site.

**Conclusion:**

Our results highlight the importance of screening for large rearrangements in CF cases with no or only one *CFTR* mutation defined.

## 1 INTRODUCTION

With the discovery of the *CFTR* (Cystic Fibrosis Transmembrane Conductance Regulator, OMIM: 602421) gene in 1989, new perspectives for the molecular study of Cystic Fibrosis (CF, OMIM: 219700) emerged (OMIM, 2018). The gene is located at 7q31.2 and mutations in both *CFTR* alleles lead to the fibrocystic phenotype (Kerem et al., 1989). The disease shows a wide clinical heterogeneity and affects mainly Caucasians. CF incidence in Europe ranges from 1 in 2000 to 3,000 live births (Farrell, 2000). However, its prevalence is quite variable in different populations and ethnicities.

The clinical diagnosis of CF is usually based on the presence of clinical features such as pancreatic insufficiency, pulmonary infections, and increased chloride concentration in sweat, which can be measured by the sweat test. The diagnosis is confirmed after the identification of two pathogenic variants in the patient's *CFTR* gene through molecular analysis.

To date, 2031 mutations of different types have been registered in the *CFTR* gene (Cystic Fibrosis Mutation Database – CFMDB, 2018), nevertheless only 312 of these variants are considered pathogenic according to the Clinical and Functional Translational of CFTR (CFTR2). The frequencies of these variants vary between populations and ethnic groups (CFMDB, 2018). Among the pathogenic variants, the F508del (c.1521_1523delCTT, p.Phe508del), a three nucleotide deletion (CTT) that results in the loss of a codon for the amino acid phenylalanine at position 508 of the protein, is the variant most commonly found in the general population. In some Caucasian populations, the F508del is present in one or both alleles in ~90% of cases (Riordan, 2008). In Brazil, its frequency varies from 23% to 48% (Bieger, Marson, & Bertuzzo, 2012). This may be due to the migration of different peoples, as well as to the heterogeneous gene flow within the country, occurring among Europeans, Africans, and Amerindians and resulting in a complex Brazilian genetic pool (Cabello, Cabello, Llerena, & Fernandes, 2006).

The most common variants of the *CFTR* gene are single nucleotide substitutions or small deletions and insertions. However, large rearrangements may occur, such as large duplications or deletions, many already described in the CFMDB; these alterations are not detected by traditional screening methods and require special techniques for their observation (Férec et al., 2006). Currently, several methods are available for the screening of genetic rearrangements, such as Multiplex Ligation‐dependent Probe Amplification (MLPA) (Schrijver, Rappahahn, Pique, Kharrazi, & Wong, 2008; Taulan et al., 2012). This methodology has been widely applied in a variety of clinical situations and investigations to detect gains or losses of genomic sequences. Therefore, we used this technique to identify large duplications or deletions in the *CFTR* gene in CF patients from the Rio de Janeiro State (Southeast Brazil), in order to characterize alleles not yet identified by conventional techniques.

## 2 MATERIAL AND METHODS

### 2.1 Ethical Compliance

The study protocol was carried out in accordance with the Declaration of Helsinki (1964) and approved by the Ethics and Research Committee of the Oswaldo Cruz Foundation (CAEE: 55095316.4.0000.5248/Protocol No: 2.010.565/17). All participants provided written authorization prior to the start of the study.

### 2.2 Patients and samples

The sample used in this study consists of 165 CF patients recruited in reference centers of Rio de Janeiro. Inclusion criteria were positive sweat test (> 60mEq) with clinical characteristics compatible with CF and only one or no defined allele after our routine screening for point mutations.

### 2.3 DNA extraction

Genomic DNA was extracted from peripheral blood leukocytes using the Purelink Genomic DNA Kit (Invitrogen, Carlsbad, CA), following the recommendations and protocol of the manufacturer.

### 2.4 MLPA Assay

The deletions/insertions were investigated with the panel assay P091‐C1 (batch 0809) (MRC‐Holland, Amsterdam, Netherlands) following the manufacturer's recommendations and protocol, except for a change in denaturation time which was increased from 5 to 10 min. DNA quantification of the samples was performed with the Qubit® 2.0 fluorometer (Applied Biosystems, Life Technologies Corporation, Carlsbad, CA). Reactions were analyzed on the ABI‐3730xl Genetic Analyzer (Applied Biosystems, Foster City, CA) in the Genotyping and Microsatellite Platform at Oswaldo Cruz Foundation/FIOCRUZ using GeneScan 500 ROX dye Size Standard (Applied Biosystems, Foster City, CA). The analysis was performed in GeneMarker® 1.9 software (SoftGenetics, LLC, State College, PA). Relative copy number (RCN) values of the *CFTR* gene (NM_000492.3) exons were calculated for each patient. We considered normal those results with values between 0.8 and 1.2. RCN values above 1.4 were considered duplications and values below 0.6 were considered deletions.

### 2.5 Real‐time quantitative PCR

Real‐time quantitative PCR with ΔΔCt method was used to confirm the variations in genic dosage detected by the MLPA technique. All samples were analyzed in triplicates. In the reaction, 4.5 pmol of each primer (available upon request) and Fast Sybr Green master mix (Applied Biosystems, Life Technologies Corporation, Carlsbad, CA) were used with 20–30 ng of DNA. The reference gene used was *Albumin* (*ALB*) (primers available upon request) and the assay was run in a StepOne Plus Real Time PCR System (Applied Biosystems, Foster City, CA). Data were analyzed by the software 7500 v2.0.6 (Applied Biosystems, Life Technologies Corporation, Carlsbad, CA).

### 2.6 Sanger Sequencing

Point mutations and polymorphisms can disrupt the hybridization site of the MLPA probes and produce a false deletion in the quantitative analysis. For this reason, samples that showed deletions in the MLPA assay were submitted to sequencing. The reaction was prepared according to the manufacturer's protocol using the Big Dye Terminator v3.1 Kit (Applied Biosystems, Foster City, CA). All reactions were processed on the ABI Prism 3130 automatic sequencer (Applied Biosystems, Foster City, CA) in the DNA Sequencing Platform at Oswaldo Cruz Foundation/ FIOCRUZ, Brazil. Sequence analysis was performed with Chromas Lite 2.0 software (Technelysium) and BioEdit Sequence Alignment Editor v6.0.6 (Isis Pharmaceuticals, Inc.).

### 2.7 In silico analysis

Three algorithms were used to analyze the functional effects of missense mutations: CADD (Rentzsch, Witten, Cooper, Shendure, & Kircher, 2018), Polyphen‐2 (Adzhubei et al., 2010) and SIFT (Sim et al., 2012).

## 3 RESULTS

Five quantitative changes in the relative number of exon copies in the *CFTR* gene were observed in five probands. Two deletions, detected on exon 2 and exon 5, were caused by small sequence variations within the hybridization region of the probe that may have compromised the probe annealing. One variation is a novel point mutation in exon 5 that causes the change of a serine to leucine at the position 168 of the protein, S168L (c.503C>T, p.Ser168Leu). The other mutation was a small deletion of 18 base pairs in exon 2 ‐ 232del18 (c.100_117delTTGTCAGACATATACCAA, p.Leu34_Gln39del).

Three copy number variations (CNVs) in the *CFTR* gene detected by MLPA were validated through quantitative Real Time PCR, an exonic duplication and two exonic deletions. One patient was found with a deletion of exons 25 and 26, CFTRdel25‐26 (NM_000492.3):c(4082+1_4081‐1)_(4418+1_4419‐1)del. We have also observed a large deletion that begins at exon 25 and extends beyond exons 26 and 27 to the next gene, *CTTNBP2* (OMIM: 609772) (CFTRdel25‐27CTTNBP2) ‐ (NM_000492.3):c(4082+1_4081‐1)_(4573+1_4575‐1)del, never described in the literature. The only duplication detected affects exons 2 and 3 (CFTRdup2‐3) NM_000492.3:c(44+1_45‐1)_(449+1_450‐1)dup and is also novel (Table 1). Finally, the F508del mutation, a three base deletion tested by one of the MLPA probes that is specific to this mutation, was observed in 34 individuals, validating our previous screenings.

**Table 1:** Large rearrangements and mutations in the *CFTR* gene identified in five probands by MLPA

Patient	Genotype	Gender	Ancestry^b^	Age of diagnosis	mEq^c^	Pancreas	Lung^e^	MLPA probe	MLPA relative copy number	qPCR 2^−ΔΔCt^
FC337^a^	F508del/CFTRdup2−3	F	African	2 years 6 months	87	PI	*Pa*	CFTR_ex02	1.448	1.735
								CFTR_ex03	1.507	1.5037
FC481^a^	F508del/CFTRdel25−26	F	Latin European	2 days	60	PI	*Pa*	CFTR ex25	0.447	0.4739
								CFTR_ex26	0.546	0.4176
FC531	A559T‐R117H/CFTRdel25−27, CTTNBP2	F	Unknown	1 years 11 months	78	PI	*Pa*	CFTR_ex25	0.480	0.4397
								CFTR_ex26	0.509	0.2993
								CFTR_ex27	0.559	0.4787
								CTTNBP2	0.468	0.4910
FC423	F508del/232del18	M	African	2 years	94	PI	*Pa*	CFTR_ex2	0.520	Na
FC383	G85E/S168L	F	African	10 months	83	PI	*Pa*	CFTR_ex5	0.682	Na

Note

PI, pancreatic insufficiency; Na, not applicable.

a Dead.

b Ancestry as informed by the patient.

c mEq ‐ Cl^‐^ dosage in the sweat.

Predictor of pulmonary function: *Pa* ‐ *Pseudomonas aeruginosa*

In silico analyzes by SIFT, PolyPhen‐2, and CADD showed that the S168L mutation is predicted as pathogenic. For the PolyPhen‐2 algorithm the score assigned was 1.00 (Pathogenicity threshold >0.8), for SIFT was 0.01 (Pathogenicity threshold <0.05) and for CADD the score was 24.9 (Pathogenicity threshold >20).

All five mutated patients presented a severe phenotype related to pancreatic insufficiency, a marker of mutation severity, and lung colonization by *Pseudomonas aeruginosa*, considered a predictor of lung function deterioration (Farrel et al., 2005; Grosse et al., 2004). One child with a duplication (FC337) ‐ CFTRdup2‐3—and other with a deletion (FC481) ‐ CFTRdel25‐26, both in combinations with the F508del mutation, died early at the age of 11 and 9 years, respectively. DNA analysis of the mothers of these patients showed that none presented the observed CNVs; they presented the F508del mutation that was inherited by their children. Unfortunately, only their mothers were available for testing.

## 4 DISCUSSION

Almost 30 years after the discovery of the *CFTR* gene, new variants continue to be identified, leading to a steady increase in the number of mutations in the gene. However, despite extensive screening, many mutations remain unknown, especially in multiethnic populations (Sosnay, Raraigh, & Gibson, 2016).

The use of the MLPA technique in our study was effective for the discovery of large deletions and duplications. It was also able to find point mutations that occurred within the probe region, interfering in their hybridization and generating a reduction of signal that can be misinterpreted as an apparent deletion. It is important to highlight that cases of deletions in a single exon, may actually represent the absence of probe binding due to the presence of a polymorphism or a point mutation. In our case, the novel mutation S168L is a clear example of a false‐positive deletion detected by the MLPA method. The involvement of a single probe requires sequencing of the hybridization target in order to exclude false‐positive findings caused by small mutations in the probe binding site. The prior verification of the existence of point mutations or polymorphisms located at the probe binding site may help to reduce false positives in the results (Schouten et al., 2002; Stuppia, Antonucci, Palka, & Gatta, 2012). PCR contamination, even in minimal amounts, may also contribute to the occurrence of false positives. Measures to avoid sample contamination are essential. This quality control is even more complicated in the hybridization process in MLPA. For this reason, the validation of the detected CNVs by other methods is essential.

In Brazil the most frequent mutations in *CFTR* are F508del, G551D (c.1652G>A, p.Gly551Asp), G542X (c.1624G>T, p.Gly542X), and R553X (c.1657C>T, p.Arg553X) (Bernardino et al., 2000; Cabello et al., 1999; Streit, Burlamaque‐Neto, de Abreu e Silva, Giugliani, & Saraiva Pereira, 2003). The Brazilian population is the product of the miscegenation of three ethnic groups: Europeans, Africans, and Amerindians. The Europeans were predominantly represented by Portuguese, although during the 16th and 17th centuries there was the immigration of French and English merchants, as well as the attempts of foreign colonization by the French in the states of Rio de Janeiro and Maranhão and by the Dutch in the North and Northeast (Salzano & Freire‐Maia, 1967). An important contribution to Brazilian ethnic admixture was the more recent migration to Brazil of Germans, Italians, Poles, Arabs, Jews, and Japanese, among others (Ramos, 1962; Salzano & Freire‐Maia, 1967). In addition, the intense gene flow between these different groups made Brazil an extremely heterogeneous country, in such a way that it can be characterized as a unique and highly complex mixture of ethnic groups.

Considering that Brazil is a continental country with a population constituted from the migration of different peoples (Krieger et al., 1965; Pena et al., 2011), we observed that the frequencies of these mutations vary considerably between the different regions of the country. For example, among the Brazilian states, we can observe differences in the frequency of F508del. In São Paulo, the frequency is around 40% (Bieger et al., 2012), in Minas Gerais 48% (Perone, Medeiros, Castillo, Aguiar, & Januário, 2010; Raskin et al., 2008), Pará, 23% (De Araújo et al., 2005), and in Rio de Janeiro, ~30% (Cabello et al., 2005; da Silva Martins et al., 2014), different from the frequency observed in Caucasian Europeans that can be of up to 90% (Riordan, 2008). Despite all the efforts to identify new mutations circulating in the country, a large number of variants remain unknown. For this reason, using diagnostic tests applied in European populations is inefficient, leading to a low detection rate. For such populations, Next Generation Sequencing (NGS) and MLPA are used nowadays as a second diagnostic alternative.

In other regions of the world, ~2% of registered *CFTR* mutations are large deletions or duplications that affect from single exons to even the entire gene (Taulan et al., 2012). Still, there are no studies showing the presence, frequency, and impact of these large deletions or duplications in the *CFTR* gene in the Brazilian population.

In the present study, we found two large deletions, the mutation CFTRdel25‐26, described in the CFMDB, and CFTRdel25‐27CTTNBP2, not described. Both are deletions that cause the loss of the terminal portion of the protein, in the nucleotide binding domain (NBD2). The CFTRdel25‐26 deletion was previously found by Hantash et al. (2006) but there are no studies showing its consequence on the protein. Since NBD2 is a critical domain to CFTR function, we believe that a large deletion involving this domain may results in a protein that is either recognized as abnormal and degraded or its conductivity is reduced. The patient FC481 had this CNV in combination with F508del. She had a clinical diagnosis determined at 2 days of age, since she had meconium ileus at birth and the immuno‐reactive trypsin test presented the altered concentration of 133ng/ml (cut off 90ng/ml). Her lungs were frequently colonized by *Pseudomonas aeruginosa* and *Staphylococcus aureus*, and the patient also had pancreatic insufficiency. The patient died at the age of nine years.

CFTRdel25‐27CTTNBP2, identified in the patient FC531, in combination with A559T (c.1675G>A, p.Ala559Thr) and R117H (c.350G>A, p.Arg117His), is a deletion that begins in exon 25 and reaches the next gene flanking the *CFTR* gene in 3’, *CTTNBP2* (Cortactin Binding Protein 2, MIM: *609772). This gene has 23 exons and is highly expressed in the brain. The patient FC531 presented a severe pulmonary condition, with chronic colonization by *Pseudomonas aeruginosa*, pancreatic insufficiency and positive sweat test with a high chloride concentration in the sweat (78 mEq/l). The diagnosis was made when she was one year and 11 months old. Her mother presented the A559T mutation. Since we could not collect DNA from the father, we assume that the patient inherited the complex allele R117H/CFTRdel25‐27CTTNBP2 from the paternal side. The patient's severe clinical history lead us to believe in a deleterious effect caused by the deletion of exons 25–27. However, additional studies are needed to better understand this finding.

In addition, we found a large in‐frame duplication of exons 2 and 3, which is involved in the formation of the N‐terminal region. It has been observed that either duplications or deletions of exon 2 reduce the amount of the produced protein, affecting both the process of transcription and transcript stability. In the case of duplications, the production of a more glycosylated protein was also observed (Taulan, 2012). The patient FC337 that presented this duplication, died at the age of 11 years. She had a history of hospitalizations, high chloride concentration in sweat (87 mEq/l), intestinal manifestations, pancreatic insufficiency, and severe pulmonary involvement. Although the variation affects a domain with minimal structural modifications and is in frame, we believe it is in trans with the F508del mutation and that this combination of alleles is responsible for the patient's severe phenotype.

Finally, we detected an apparent deletion caused by the point mutation S168L in exon 5, which lies exactly in the probe hybridization site and completely disrupts the affinity of the probe to the site, generating a low signal, interpreted as a deletion. The patient FC383 has this mutation in combination with G85E (c.254G>A, p.Gly85Glu). Genetic tests have shown that this missense mutation in exon 5, registered as rs869249241 in dbSNP, was transmitted by the mother to the CF patient, and although the father's DNA has not been collected for analysis, it is possible that the other allele (G85E) has been inherited from him. The patient was diagnosed at 10 months of age, she had a history of recurrent pneumonia and a sweat test of 83 mEq/l. She presented severe pulmonary involvement, with colonization by *Pseudomonas aeruginosa* and *Staphylococcus aureus,* and pancreatic insufficiency. We used three algorithms for the prediction of the impact of the S168L mutation on protein function. All of them predicted the mutation to be deleterious. This mutation was also validated by new generation sequencing.

It is important to mention that of the five patients with the variants identified in this study, three have African ancestry, one has Latin European ancestry and one is unaware of the origin of the grandparents. In addition, the three mutations, G85E, A559T and R117H, in heterozygosis with the rearrangements found in this work, are part of the African Diaspora as observed in the study by Stewart and Pepper (2017).

The use of the MLPA assay, the automatic sequencing and the Quantitative PCR to support and validate the presence of *CFTR* rearrangements in our patients allowed us to increase the rate of detection of unknown alleles. Our study is a pioneer in Brazil and shows that, after an exhaustive sequence analysis of our patients, the screening of large deletions and insertions should be integrated into the mutation analysis strategy for patients with suggestive clinical presentation and no identified mutation by conventional tests.

## 5 CONCLUSION

The identification of new pathogenic variants is very important for the study of CF in Brazil, since we still lack information about the mutations that circulate in this population. This study shows that there is still much to be explored and that there are several and unknown variants acting in the development of CF in Brazil, whose multi ethnicity decreases the efficiency of European genetic tests. In addition, the results obtained in this work also show the importance of recommending the analysis of CNVs in the *CFTR* gene in the Brazilian population and in other multiethnic populations.

## CONFLICT OF INTEREST

The authors declare that there is no conflict of interests.
